# Myocardial Fibrosis in Athletes: Risk Marker or Physiological Adaptation?

**DOI:** 10.3390/biomedicines13112747

**Published:** 2025-11-10

**Authors:** Vasiliki Katsi, Epameinondas Triantafyllou, Christos Fragoulis, Christos Vazaios, Spyridon Maragkoudakis, Alexandros Kasiakogias, Charalampos Vlachopoulos, Konstantinos P. Tsioufis

**Affiliations:** 1 First Department of Cardiology, Hippokration General Hospital, National and Kapodistrian University of Athens Medical School, 114 Vasilissis Sofias Avenue, 11527 Athens, Greece; christosfragoulis@yahoo.com (C.F.); akasiakogias@gmail.com (A.K.);; 2 Department of Thoracic Surgery, General Oncological Hospital of Peiraeus, 18537 Pireas, Greece; vazaios.christos@gmail.com; 3 General Hospital of Chania Cardiology Department, 73300 Chania, Greece

**Keywords:** exercise, athletes, myocardial fibrosis, risk stratification, cardiac magnetic resonance, ventricular scar

## Abstract

Endurance exercise is widely recognized for its cardiovascular benefits, including improved longevity and metabolic health. However, excessive endurance training may lead to adverse cardiac adaptations, such as myocardial fibrosis, detected via late gadolinium enhancement (LGE) on cardiac magnetic resonance imaging (CMR). This review examines the dual role of myocardial fibrosis in athletes—as a potential risk marker for life-threatening arrhythmias or a benign byproduct of physiological remodeling. While moderate exercise promotes beneficial cardiac hypertrophy, ultra-endurance athletes exhibit a 10–20% increase in ventricular size and mass, alongside elevated cardiac biomarkers post-exercise. Myocardial fibrosis, particularly in the left ventricle (LV), is associated with arrhythmias and sudden cardiac death, especially when presenting as a subepicardial/midmyocardial patchy pattern. Studies report that 22% of athletes with this pattern experienced malignant arrhythmias, underscoring its clinical significance. Conversely, fibrosis may also reflect adaptive remodeling in some cases, complicating its interpretation. The mechanisms underlying fibrosis in athletes remain unclear but may involve repeated cardiac stress, inflammation, or distinct atherosclerotic plaque dynamics. CMR is critical for detecting fibrosis, though differentiating pathological from physiological patterns requires careful clinical correlation. Risk stratification must consider LGE patterns, arrhythmia history, and symptoms. Despite concerns, elite athletes generally exhibit increased longevity, highlighting the complex interplay between exercise benefits and risks. Further research is needed to clarify fibrosis mechanisms, refine diagnostic criteria, and guide management strategies to ensure athlete safety while preserving the advantages of endurance training.

## 1. Introduction

Exercise of moderate intensity is associated with improved health outcomes and a significant increase in longevity. The benefits of exercise extend across various physiological systems, contributing to enhanced cardiovascular function, metabolic health, and overall well-being. Some studies even suggest a dose–response relationship, indicating that greater amounts of exercise correlate with greater health benefits [1,2,3,4,5]. This concept has led to the popularity of endurance sports, where athletes often push the boundaries of human performance.

Endurance athletes exceed the recommendations for exercise for the general population by a substantial margin. This often could reach a 15-fold to 20-fold higher workload volume [2,5]. This high level of activity places significant stimulus on the cardiovascular system, thus prompting notable physiological adaptations. Specifically, the heart undergoes remodeling in order to accommodate the sustained need for higher cardiac output over prolonged periods. These adaptations include a 10–20% increase in both left and right ventricular size and a substantial increase in left ventricular mass [2,5,6].

While the positive effects of exercise are well-known and accepted, there is a new dilemma regarding the probable consequences of ultra-endurance exercises over a long period of time. Some studies suggest that there may be a reduction in cardiovascular benefit for individuals with high amounts of endurance exercise [3]. Several observations support a higher prevalence of cardiac changes among high-intensity and master athletes, including high coronary artery calcium (CAC) scores, atrial arrhythmias, and the presence of myocardial fibrosis [3].

The purpose of this review is to investigate the current status regarding the clinical significance and management of documented myocardial fibrosis among athletes practicing endurance sports and high-intensity exercise. Little remains understood about pathophysiological mechanisms leading to this condition, and its relevance to athletes is unknown. There is even contention as to whether the condition serves chiefly as a risk marker for unfavorable cardiac events or as a byproduct of intense training.

## 2. Methodology

A narrative literature review was conducted to explore myocardial fibrosis in endurance athletes, with the aim of summarizing current knowledge on its pathophysiological mechanisms, clinical relevance, and role in risk stratification when detected by (CMR). A comprehensive search of the Medline database was carried out up to September 2025 using the following keywords and Boolean operators: “Myocardial fibrosis,” “Athletes,” “Cardiac adaptation,” “Athlete’s heart,” “Exercise and cardiac adaptations,” “Non-ischemic heart disease,” and “Physiology of the heart.” Only English-language publications from January 2000 onward were included. The search initially identified about 121 articles. Titles and abstracts were screened for relevance, with inclusion limited to studies addressing myocardial fibrosis or cardiac remodeling in endurance athletes. Exclusion criteria were studies not focused on myocardial fibrosis, those involving non-athlete populations, or reviews lacking original data. Full-text screening was then performed to select articles offering clinical, imaging, or mechanistic insights aligned with the review’s objectives. The review incorporated observational and interventional studies, as well as systematic reviews and meta-analyses examining myocardial fibrosis in athletes. Case reports and small case series were also considered when they provided illustrative insights. Studies outside the context of endurance sports or fibrosis assessment by late gadolinium enhancement on CMR were excluded. Although the review does not adhere to a formal systematic review protocol, efforts were made to prioritize recent and methodologically sound research to ensure a balanced overview. No formal quality scoring was applied, consistent with narrative review methodology. The final selection sought to capture studies that enhance understanding of the mechanisms, imaging patterns, clinical implications, and management strategies related to myocardial fibrosis in endurance athletes.

## 3. Cardiac Fibrosis: An Overview

Fibrosis, in general, involves the pathological remodeling of the extracellular matrix (ECM) [3]. In the heart, fibrosis is often the result of diverse processes including aging, injury, or underlying myocardial disease. This pathological remodeling ultimately leads to the formation of fibrotic scars within the myocardium [7]. Fibrotic scars can profoundly affect the physiological function of the heart-anatomically, mechanically and electrically. They can impair the heart’s contractility leading to reduced ejection fraction (EF). This is due to the fact that the fibrotic tissue is not functional and also stiffens the myocardial wall [7]. Additionally, fibrotic tissue can disrupt the heart’s electrical conduction system, leading to arrhythmias, This arrhythmogenic activity has been considered as one of the potential mechanisms of sudden cardiac death observed in patients with cardiomyopathies [7]. It is important though to understand that cardiac fibrosis may be a complex entity. There are different patterns of cardiac scars, each with its own distinct characteristics and underlying cause. Understanding these different types is crucial for accurate diagnosis, prognosis, and management of cardiac conditions. Furthermore, reparative (replacement) fibrosis represents the deposition of collagen in areas of myocyte necrosis or focal injury, forming discrete scar tissue that may disrupt myocardial conduction and increase arrhythmic risk. In contrast, interstitial fibrosis refers to diffuse collagen accumulation between viable myocytes, typically resulting from chronic mechanical stress or neurohormonal activation. In endurance athletes, limited interstitial fibrosis may reflect a reversible adaptive response, whereas reparative fibrosis—especially if patchy or midmyocardial—has been associated with a higher risk of arrhythmias and adverse outcomes.

## 4. Exercise-Induced Cardiac Remodeling

Endurance training refers to sustained, high-volume aerobic exercise performed at moderate intensity (typically 60–80% of maximal heart rate) for prolonged durations, often exceeding five hours of vigorous activity per week or 8–10 METs per session, as seen in long-distance runners, cyclists, and triathletes. The above mentioned, provokes significant cardiac remodeling, which is generally considered as a physiological adaptation to the increased demands placed on the heart. However, there is a growing interest in whether certain types or intensities of exercise may lead to maladaptive remodeling in predisposed individuals [8]. One area of focus is the potential for arrhythmogenic cardiac remodeling, particularly involving the right ventricle (RV) [8]. The RV, with its thinner walls and unique hemodynamic profile, may be more susceptible to the effects of long-term, intense endurance exercise. Studies have investigated whether intense endurance exercise disproportionately affects the RV compared to the left ventricle (LV) and at the same time whether the exposure to endurance competition influences cardiac remodeling, including the development of fibrosis, in well-trained athletes [8,9]. Following vigorous endurance events, it’s common not only to observe signs of borderline cardiac dysfunction but also elevated levels of cardiac biomarkers. These biomarkers, such as troponins [10] and B-type natriuretic peptide (BNP), are typically released into the blood serum in response to cardiac injury or stress. The clinical meaning of elevated biomarkers—especially troponin—after exercise is still uncertain. These elevations show that the heart has been under stress, but it is not yet clear whether they reflect a temporary, reversible physiological response or evidence of actual injury. This distinction remains an active area of debate and investigation [5,10,11].

## 5. Myocardial Fibrosis in Athletes

Myocardial fibrosis, which can be identified using (LGE) on cardiac magnetic resonance imaging (CMR), has been observed in a subset of endurance athletes [1]. The detection of LGE in athletes raises important questions about its significance and whether it can sometimes represent a benign adaptation or may be an indication of adverse cardiac outcomes. The interpretation of myocardial fibrosis in the athletic population is complex and requires careful consideration of various factors, including the location, pattern, and extent of fibrosis, as well as the athlete’s clinical presentation and other relevant findings [1,7].

**Figure 1:**
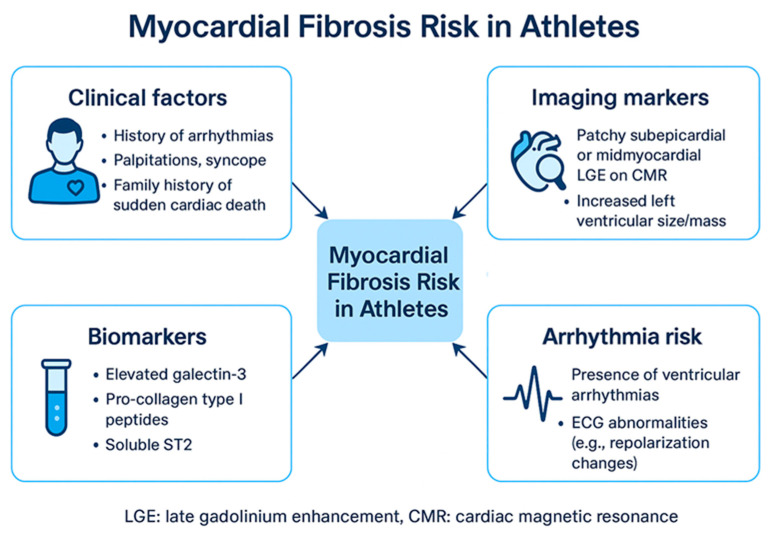
Myocardial Fibrosis Risk in Athletes. ECG: Electrocardiogram, LGE: late gadolinium enhancement.

**Figure 2:**
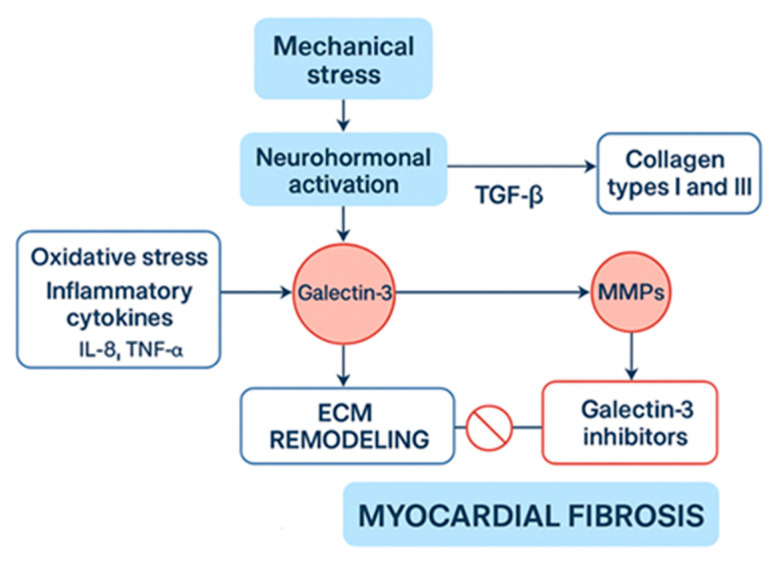
Physiological Pathways and Interventions in Myocardial Fibrosis. ECM: Extracellular matrix, IL-8: Interleukin-8, MMPs: matrix metalloproteinases, TGF-a/β: transforming growth factor-alpha/beta.

Myocardial fibrosis in athletes may arise from a complex interaction of mechanical stress, neurohormonal activation, and the mismanagement of extracellular matrix (ECM) remodeling. Repeated hemodynamic overload during strenuous exercise elevates myocardial wall stress, which activates cardiac fibroblasts and stimulates collagen deposition (primarily types I and III) through transforming growth factor-beta (TGF-β)/Smad signaling [12]. Pro-collagen type I is the principal precursor of mature collagen fibrils. Its upregulation, driven by TGF-β and mechanical stretch, enhances myocardial stiffness by increasing collagen cross-linking and reducing compliance. Elevated circulating levels of pro-collagen I peptides have been correlated with both pathological and exercise-induced myocardial fibrosis, serving as a potential biomarker for early extracellular matrix remodeling. In cases of physiological adaptation, temporary ECM remodeling improves myocardial stiffness and efficiency, facilitated by a balanced activity of matrix metalloproteinases (MMPs) and tissue inhibitors of metalloproteinases (TIMPs) [13,14]. However, when stress is excessive or prolonged, this balance can tip towards pathological fibrosis, which is marked by excessive collagen cross-linking and decreased degradation due to heightened levels of TGF-β, angiotensin II, and pro-fibrotic factors such as connective tissue growth factor [15]. Furthermore, oxidative stress and inflammation, instigated by NADPH oxidases and inflammatory cytokines (e.g., IL-6, TNF-α), worsen fibroblast activation and ECM accumulation [16]. Distinguishing between adaptive remodeling and maladaptive fibrosis depends on whether collagen deposition can be reversed and the extent of persistent fibroblast activation, commonly evaluated through biomarkers such as galectin-3 and soluble ST2 [17]. Recent research indicates that endurance athletes participating in extreme training regimes may show signs of subclinical fibrosis, which can be identified through CMR, raising concerns about potential long-term risks for arrhythmias [18,19,20]. Emerging biomarkers such as galectin-3 and soluble ST2 reflect myocardial stress and extracellular matrix turnover. Elevated galectin-3 levels have been linked to persistent fibroblast activation, while soluble ST2 reflects stretch-induced signaling associated with adverse remodeling. Their longitudinal assessment may provide valuable insight into dynamic changes in myocardial fibrosis among endurance athletes (Table 1, Figure 1 and Figure 2).

Endurance athletes tend to show a higher occurrence of atrial fibrosis, which has been closely linked to a greater risk of developing atrial arrhythmias, particularly atrial fibrillation (AF). This relationship appears to stem from the structural and electrical changes the atria undergo in response to prolonged exercise. Specifically, extended periods of endurance training promote enlargement of the atria and increased mechanical stress on their walls, encouraging the buildup of fibrotic tissue within the atrial muscle. This fibrosis disrupts the heart’s normal electrical pathways, leading to conditions favorable for irregular heart rhythms. Additionally, endurance exercise influences the autonomic nervous system, increasing vagal tone, which shortens the refractory period in the atria and further predisposes to AF. Research in elite endurance athletes, including long-distance runners and rowers, consistently shows a significantly higher rate of AF compared to non-athletic individuals, highlighting the clinical relevance of these changes. Newer studies also point to differences between sexes in how susceptible they are to these effects, as well as suggest that fibrosis and arrhythmias might partially reverse with reduced training. This growing body of evidence underscores the complex balance athletes face: while exercise offers numerous cardiovascular benefits, it may paradoxically elevate AF risk, thereby warranting careful clinical surveillance and personalized management plans in this population [21,22].

## 6. Left Ventricular Scar

One study provided valuable insights into the potential implications of LV scar in athletes [1]. This study compared three groups of athletes: those with ventricular arrhythmias and isolated LV subepicardial/midmyocardial LGE, those with ventricular arrhythmias but no LGE, and a group of healthy control athletes [1,23]. The researchers observed that in the first group LGE predominantly involved the lateral LV wall, in 77% of athletes with LV scar. This pattern was not observed in any of the athletes in the control group (*p* < 0.001). Athletes exhibiting this patchy pattern often presented with ventricular arrhythmias characterized by a predominant right bundle branch block morphology. Additionally, a significant proportion of these athletes showed electrocardiogram (ECG) repolarization abnormalities (48%), and some demonstrated echocardiographic evidence of hypokinesis of the lateral LV wall (19%) [1]. The most alarming finding was the association between the patchy LGE pattern and adverse arrhythmic events. During a follow-up period of 38 ± 25 months, 6 of 27 (22%) athletes with the aforementioned pattern experienced malignant arrhythmic events, including appropriate implantable cardiac defibrillator (ICD) shock (n = 4), sustained ventricular tachycardia (n = 1), or sudden death (n = 1). In contrast, none of the athletes without LGE or those with a spotty LGE pattern, nor any of the control athletes, experienced such events [4]. These findings highlight the potential clinical significance of isolated non-ischemic LV LGE with a patchy pattern in athletes. The study concluded that this specific pattern of fibrosis may be associated with an increased risk of life-threatening arrhythmias and sudden death in the athletic population. Furthermore, the subepicardial/midmyocardial location of this type of LV scar often makes it difficult to detect using traditional echocardiography, emphasizing the importance of CMR for accurate assessment [1,4].

## 7. Coronary Artery Disease and Myocardial Fibrosis

To provide further context, it’s helpful to consider the role of myocardial fibrosis in individuals with coronary artery disease (CAD). In patients with CAD, contrast-enhanced CMR is a valuable tool for identifying myocardial scar resulting from myocardial infarction (MI). Numerous studies have investigated the prognostic implications of unrecognized myocardial scar detected by CMR in patients without a documented history of MI [9,19]. One such study assessed 195 patients with suspected CAD but no known prior MI who underwent CMR. During a median follow-up period of 16 months, 18% of these patients experienced major adverse cardiac events (MACE), listing: [(1) cardiac death, (2) new acute MI, (3) unstable angina requiring hospitalization, (4) development or progression of heart failure requiring hospitalization, or (5) ventricular arrhythmias requiring appropriate discharge from an internal cardioverter/defibrillator (ICD)] [23], including 17 deaths. LGE demonstrated strong unadjusted associations with MACE and cardiac mortality, with hazard ratios of 8.29 and 10.9, respectively (both *p* < 0.0001) [23]. Notably, patients in the lowest tertile of LGE-involved myocardium (mean LV mass, 1.4%) still experienced a greater than 7-fold increased risk for MACE [9,19]. In multivariable analyses, LGE remained independently associated with MACE, even after accounting for other clinical factors, angiographic findings, and measures of LV function [4]. LGE was the strongest predictor selected in the best overall models for both MACE and cardiac mortality. The study concluded that in patients with suspected CAD but no history of MI, the presence of LGE, even involving a small amount of myocardium, carries a significant cardiac risk. Furthermore, LGE provides additional prognostic value for predicting MACE and cardiac mortality beyond that offered by common clinical, angiographic, and functional predictors [9,19].

## 8. Potential Mechanisms of Fibrosis and Coronary Artery Calcium Development in Athletes

The mechanisms that underlie the development of myocardial fibrosis in athletes are not fully explained. It is believed that these mechanisms may differ from those observed in sedentary individuals. One study [9] investigated this theory by examining elite athletes with a low atherosclerotic risk profile. The researchers found that male athletes had a significantly higher prevalence of atherosclerotic plaques compared with sedentary males [9] (44.3% versus 22.2%; *p* = 0.009) [23]. Remarkably, the characteristics of these plaques also differed between the two groups. Male athletes predominantly exhibited calcific plaques (72.7%), which are generally considered more stable, while sedentary males showed predominantly mixed morphology plaques (61.5%), which may be more prone to rupture [9,19]. The study also identified a correlation between the number of years of training and the risk of increased CAC. The number of years of training was the only independent variable associated with an increased risk of CAC > 70th percentile for age or luminal stenosis ≥ 50% in male athletes (odds ratio, 1.08; 95% confidence interval, 1.01–1.15; *p* = 0.016). These findings suggest that the pathophysiology of plaque formation in athletes may indeed differ from that in sedentary individuals. While coronary plaques appear to be more abundant in athletes, their predominantly stable nature could potentially moderate the risk of plaque rupture and acute myocardial infarction [9,19]. The relationship between elevated CAC and myocardial fibrosis is complex and not direct. CAC reflects atherosclerotic burden and develops through mechanisms that differ from those driving myocardial fibrosis. In athletes, higher CAC scores often represent stable calcified plaques, which may not indicate active fibrosis or ischemic injury. It is therefore important to clarify that any observed correlation between CAC and myocardial fibrosis should not be interpreted as causative. Rather, it may be coincidental, and further research is needed to better understand the potential links between these processes [18].

## 9. Cardiovascular Health and Cardiorespiratory Fitness

Regular physical exercise offers a multitude of well-established health benefits and plays a vital role in combating obesity and its associated cardiovascular complications. Exercise is not only a preventive measure against the onset and development of cardiovascular disease but also an important therapeutic tool for improving outcomes in patients with existing cardiovascular conditions [3,5,23]. The beneficial effects of exercise on the cardiovascular system are wide-ranging. These include enhanced mitochondrial function, restoration and improvement of vasculature, and the release of myokines from skeletal muscle, which contribute to preserving and augmenting cardiovascular function. Exercise also improves overall metabolic health, reduces inflammation, decreases the risk of heart failure, and improves survival rates, as mentioned before. Furthermore, it improves glucose tolerance, insulin sensitivity, and lipid concentrations, all of which contribute to a healthier cardiovascular profile [3]. Regular exercise can also lead to a decrease in resting heart rate, blood pressure, and levels of atherogenic markers, while promoting physiological cardiac hypertrophy, a beneficial adaptation to the demands of exercise. Additionally, exercise enhances myocardial perfusion and increases high-density lipoprotein cholesterol levels, both of which contribute to reducing stress on the heart [10,24]. Higher levels of cardiorespiratory fitness, which serve as an indicator of regular physical activity, are consistently associated with a lower risk of cardiovascular disease mortality [25]. This protective effect of fitness is observed even in individuals who are overweight or obese and those with diabetes, highlighting the powerful benefits of exercise across various populations [25,26].

## 10. Endurance Exercise: Strength or Stress?

While the benefits of regular aerobic exercise of moderate intensity are widely established and accepted, the effects of high-volume endurance exercise are more complex and under investigation. Endurance athletes, who frequently push the limits of exercise volume and intensity, may experience a combination of both physiological adaptations and potential maladaptations [25,26]. The sustained high cardiac output demanded by endurance exercise leads to significant cardiac remodeling, including increases in ventricular size and mass [23,25]. As mentioned earlier, a significant proportion of endurance athletes exhibit elevated cardiac biomarkers and signs of cardiac dysfunction immediately following intense endurance events. In the long term, some endurance athletes show an increased prevalence of coronary artery disease, myocardial fibrosis, and arrhythmias. These potentially harmful adaptations raise concerns and prompt ongoing research into the potential detrimental effects of excessive endurance exercise in susceptible individuals [2,20,24]. It is crucial to acknowledge that despite these concerns and observations, elite endurance athletes, as a group, generally have increased life expectancy compared to the general population. Regular aerobic exercise provides robust protection against atherosclerotic cardiovascular disease, certain types of malignancies, and age-related disability, ultimately contributing to increased lifespan. This highlights the complex interplay between the benefits and potential risks associated with high-volume endurance exercise [2,9,20].

## 11. Myocardial Fibrosis: Risk Marker or Exercise Induced Adaptation?

The presence of myocardial fibrosis, particularly the subepicardial/midmyocardial patchy pattern, appears to be a risk marker for life-threatening arrhythmias and sudden cardiac death in some athletes [1]. However, fibrosis may also appear as a physiological adaptation related to endurance training in other contexts. Intense endurance training appears to trigger a mild and controlled increase in interstitial fibrosis as part of the heart’s way of adapting its extracellular matrix to heightened physical demands. This subtle buildup of collagen within the myocardial interstitium likely functions to strengthen the heart’s structural framework, helping it to endure the increased mechanical stress that comes with prolonged high-intensity exercise. Research has revealed that genes linked to fibrosis, including COL3A1, and enzymes responsible for matrix remodeling, such as MMP-2 and TIMP-1, are upregulated following long-term endurance exercise [27,28]. These changes reflect an active remodeling process that aligns with physiological adaptation rather than harmful fibrosis. It is important to recognize that this form of adaptive fibrosis differs from the permanent scarring seen in disease states, though the clinical distinction is often nuanced. Distinguishing pathological from adaptive fibrosis remains a major clinical challenge requiring further research [7,19]. A critical challenge is to develop a more precise understanding of the differences between pathological fibrosis, which clearly increases the risk of adverse cardiac events, and physiological remodeling, which may occur as a benign adaptation to the demands of exercise [7]. This distinction is critical for appropriate clinical decision-making in athletes with evidence of myocardial fibrosis [7].

## 12. Diagnostic Challenges and the Role of Cardiac Magnetic Resonance Imaging

CMR has emerged as the gold standard for the non-invasive assessment of myocardial fibrosis. Its ability to provide high-resolution images of the heart and its unique tissue characterization capabilities make it invaluable for detecting and quantifying LGE, the hallmark of myocardial fibrosis [22]. CMR offers several advantages over other imaging modalities in the evaluation of athletes’ hearts. Echocardiography, while useful for assessing cardiac structure and function, often has limitations in detecting subtle or localized areas of fibrosis, particularly in the subepicardial or midmyocardial layers [4]. CMR’s superior spatial resolution and tissue contrast allow for the identification of even small regions of fibrosis that may be missed by other techniques. The use of LGE imaging in CMR is based on the principle that damaged or scarred myocardial tissue retains gadolinium-based [1,4,7] contrast agents for a longer period compared to healthy myocardium. This difference in contrast enhancement allows for the visualization and quantification of fibrotic areas. CMR can also provide valuable information about the location, pattern, and extent of fibrosis, which can be crucial for risk stratification [7]. However, the interpretation of CMR findings in athletes requires expertise and careful consideration of the athlete’s clinical context. The presence of LGE does not automatically equate to pathology, and distinguishing between physiological remodeling and pathological fibrosis can be challenging.

T1 mapping in CMR is another promising tool, by allowing quantitative assessment of myocardial tissue characteristics. By measuring native T1 relaxation times, and in combination with contrast imaging calculating extracellular Volume (ECV), it provides valuable insight into diffuse myocardial fibrosis and overall myocardial composition. In athletes, T1 mapping highlights two contrasting processes. On one hand, increased ECV can indicate diffuse interstitial fibrosis; on the other, reduced native T1 values often reflect intracellular volume expansion linked to physiological myocardial hypertrophy. This intracellular expansion corresponds to larger cardiomyocytes and greater intracellular water content, features typical of the athlete’s heart adaptation [29,30]. Sex differences are notable: female athletes tend to show higher native T1 and ECV values than males, likely due to hormonal influences on myocardial tissue [30]. Sport type also plays a role; endurance athletes, for example, often present with lower native T1 times compared to power or skill athletes, reflecting different remodeling patterns [29]. Clinically, T1 mapping is valuable because it helps distinguish between normal physiological adaptations and early signs of pathology, such as myocarditis or cardiomyopathy, supporting more tailored clinical decisions [18]. However, interpretation in athletes requires caution and should incorporate sex- and sport-specific reference ranges to minimize misclassification [30,31]. Ongoing research is essential to clarify its prognostic significance and to refine its role in cardiac screening for athletes.

Factors such as the athlete’s training history, symptoms, ECG findings, and other relevant clinical data must be accounted as well [1,7,32].

## 13. The Spectrum of Myocardial Fibrosis Patterns in Athletes

Different patterns of LGE have been observed among athletes, and these patterns may have varying clinical implications [7,8,22].

- **Patchy Pattern including the fibrotic stria:** Associated with increased arrhythmic risk and sudden cardiac death
- **Focal LGE:** Variable clinical significance depending on location and etiology; may reflect benign remodeling or potential arrhythmogenic substrate.
- **Diffuse LGE:** Rare, usually indicating underlying cardiomyopathies warranting further evaluation.

Understanding the different patterns of LGE and their clinical correlates is crucial for accurate risk stratification and management of athletes with myocardial fibrosis [7] (Table 2 and Table 3).

## 14. Risk Stratification and Management of Athletes with Myocardial Fibrosis

The management of athletes with myocardial fibrosis is a complex and evolving area. There are no definitive guidelines, and clinical decisions must be individualized based on a careful assessment of the athlete’s risk profile [2,19,20,23]. Risk stratification is a critical component of the management process. The goal is to identify athletes who are at increased risk of adverse cardiac events and to implement appropriate interventions to moderate that risk. Factors that may influence risk stratification include:

- **Pattern and Extent of LGE:** As discussed earlier, certain patterns of LGE, such as the stria pattern, have been associated with a higher risk. The extent of LGE, or the amount of myocardium involved, is also an important consideration.
- **Presence of Arrhythmias:** Athletes with a history of arrhythmias, particularly ventricular arrhythmias, are at higher risk.
- **Symptoms:** The presence of symptoms such as chest pain, palpitations, or syncope raises concern.
- **ECG Findings:** Abnormalities on the (ECG), such as repolarization abnormalities or conduction abnormalities, may indicate an increased risk.
- **Echocardiographic Findings:** Evidence of LV dysfunction or other abnormalities on echocardiography may be a sign of increased risk.
- **Exercise History and Intensity:** The athlete’s training history and the intensity of their exercise regimen may be relevant factors.

The aforementioned work-up could be used to clarify whether the myocardial fibrosis is a result of exercise or cardiomyopathy (myocarditis, arrhythmogenic etc.) that would need more intensive management (Table 3) especially for athletes with high-risk features.

High-risk features in athletes encompass clinical, imaging, and electrophysiological findings that point to a heightened likelihood of adverse cardiac events, including sudden cardiac death. Among the strongest markers are complex or unusual ventricular arrhythmias, such as polymorphic or sustained ventricular tachycardia, which are often linked to underlying structural heart disease [33].

Cardiac MRI adds further value: extensive myocardial fibrosis, especially when seen as subepicardial or mid-wall stria patterns on LGE, is considered a marker of increased arrhythmic vulnerability [34]. Symptoms like unexplained fainting or palpitations, alongside a family history of premature sudden death, heighten clinical concern [35].

According to the 2020 ESC Sports Cardiology Guidelines, the presence of LGE or ventricular arrhythmias mandates individualized evaluation. Athletes with extensive or patchy LGE should be restricted from high-intensity endurance exercise until thorough risk assessment. In contrast, those with minor or non-progressive LGE and no arrhythmias may continue moderate exercise under surveillance (Pelliccia et al., Eur Heart J 2020) [35].

Electrocardiography also plays a role. Features such as prolonged QT intervals, abnormal repolarization, or conduction delays contribute to overall risk assessment. Similarly, non-sustained ventricular tachycardia (NSVT) detected on Holter monitoring is a notable finding, particularly when combined with other risk markers [34]. Exercise testing may also reveal exertional symptoms or arrhythmias that would otherwise remain hidden [33].

Genetic testing may sometimes be offered in certain grey zone areas or when there is evidence of a specific cardiomyopathy. Identifying mutations such as those in DSP or LMNA genes can signal an inherited predisposition to arrhythmogenic cardiomyopathy and sudden death, even in athletes with no obvious structural changes [35].

Ultimately, integrating clinical history, imaging, electrophysiological data, and genetic insights allows for a nuanced, individualized approach to managing athletes. This enables clinicians to balance the demands of performance with the imperative of safety [29]. In athletes with patchy or extensive LGE and high-risk features, endurance training should be discontinued or substantially reduced, consistent with current guideline recommendations. Pharmacological therapy is not routinely indicated unless guided by the presence of symptomatic arrhythmias or underlying cardiomyopathy [33,34].

The presence of myocardial fibrosis requires individualized counseling regarding training volume and competition eligibility. Return-to-play decisions should be based on fibrosis extent, arrhythmic risk, and clinical presentation. Athletes with isolated mild LGE and no arrhythmias may safely resume training under monitoring, whereas those with patchy or extensive LGE and arrhythmic events should restrict or discontinue endurance competition until further evaluation [35].

The diagnostic and therapeutic interventions for athletes with myocardial fibrosis are presented in Table 4 and Graphical Abstract.

## 15. Future Directions

Further progress in research on myocardial fibrosis in athletes is vital to better understand the fine distinction between beneficial physiological cardiac remodeling and harmful pathological changes triggered by intense endurance training. Current data highlight the difficulty in distinguishing harmless training-related fibrosis from fibrosis that poses increased risks for severe arrhythmias and sudden cardiac death, especially when recognized by specific patterns such as the subepicardial or midmyocardial patchy on CMR. Future research should prioritize refining diagnostic criteria and advancing imaging technologies to more accurately differentiate adaptive cardiac remodeling from detrimental fibrosis. Additionally, it is important to investigate the molecular and cellular mechanisms driving fibrosis, including the contributions of mechanical stress, neurohormonal pathways, inflammation, and the regulatory interplay between MMPs and TIMPs. The identification and validation of biomarkers like galectin-3 and soluble ST2 for early detection and monitoring could improve risk prediction in athletes. Longitudinal studies examining the long-term cardiovascular effects of myocardial fibrosis in this population are also necessary. Developing personalized management approaches, including customized exercise regimens and targeted medical therapies, will be key to minimizing adverse outcomes while maintaining the cardiovascular benefits of endurance exercise. These efforts will help promote safer athletic participation and optimize heart health among athletes. Future research should prioritize multicenter, prospective cohort studies integrating CMR, biomarkers (galectin-3, ST2), and clinical outcomes. Harmonized imaging and reporting protocols are essential to enable reproducibility and meta-analytic comparison across athletic populations.

## 16. Conclusions

Endurance exercise induces significant cardiac adaptations. Some of these adaptations may result in pathological remodeling of the heart, such as myocardial fibrosis. While concerns exist regarding the potential risks associated with excessive endurance exercise, it is important to acknowledge that elite endurance athletes, as a group, demonstrate increased longevity. However, this benefit likely does not extend to individuals exhibiting patchy or extensive LGE patterns, in whom the risk of malignant arrhythmias and sudden cardiac death outweighs the expected longevity advantage. Myocardial fibrosis in athletes is a complex and multifaceted phenomenon. In certain situations, such as the presence of a patchy LGE pattern, it may serve as a risk marker for adverse events, including life-threatening arrhythmias and sudden cardiac death. In other contexts, it may represent a part of the physiological remodeling process in response to the demands of intense training. Further research is essential to determine the precise role of myocardial fibrosis in athletes, to differentiate between pathological fibrosis and physiological remodeling, and to develop appropriate clinical strategies for risk stratification, management, and treatment. A greater understanding of this complex issue will help ensure the safety and well-being of athletes while allowing them to continue to pursue the benefits of endurance exercise. Despite accumulating evidence, major knowledge gaps remain in differentiating physiological from pathological fibrosis, understanding molecular mechanisms, and defining safe exercise thresholds. Again, future research should integrate advanced imaging, biomarker validation, and genetic screening to refine risk stratification. Priority areas include prevention of maladaptive fibrosis, longitudinal athlete follow-up, and interventional trials assessing the impact of tailored exercise reduction or pharmacologic modulation.

## Figures and Tables

**Table 1:** Key differences between pathological myocardial fibrosis and physiological remodeling (“athlete’s heart”) based on imaging, biomarkers, clinical features, prognosis, and management implications.

	Pathological Fibrosis	Physiological Remodeling
**Nature**	Reparative or maladaptive collagen deposition after injury; persistent ECM expansion.	Adaptive chamber enlargement and myocyte hypertrophy; reversible.
**CMR pattern**	Patchy subepicardial or mid-myocardial LGE (often LV lateral wall); ↑ native T1/ECV.	No or minimal LGE; normal–low native T1, normal ECV.
**Biomarkers**	↑ Pro-collagen I, galectin-3, soluble ST2.	Usually normal; transient troponin/BNP rise post-exercise only.
**Function/ECG**	Possible LV dysfunction, arrhythmias, repolarization or conduction abnormalities.	Preserved function, sinus bradycardia, physiological electrocardiogram (ECG) changes.
**Prognosis**	↑ Risk of ventricular arrhythmias and SCD; irreversible.	Benign, reversible with detraining; associated with longevity.
**Management**	Restrict endurance training; evaluate for arrhythmias/ICD if indicated.	Continue training under routine follow-up; no specific therapy.

BNP: Brain Natriuretic Peptide, CMR: Cardiac Magnetic Resonance, ECG: Electrocardiogram, ECM: Extracellular matrix, ECV: Extracellular volume ICD: Implantable Cardioverter Defibrillator, LGE: Late Gadolinium Enhancement, LV: left ventricle, SCD: Sudden Cardiac Death, ↑: Increase.

**Table 2:** Patterns of myocardial fibrosis among athletes.

Fibrosis Pattern	Location	Clinical Implications
Patchy Pattern	Subepicardial/midmyocardial layers of left ventricle (LV)	Linked to increased risk of ventricular arrhythmias and sudden cardiac death; regarded as a troubling fibrosis form
Focal LGE	Variable myocardial regions	Significance varies; can be benign or related to elevated arrhythmia risk depending on location and cause
Diffuse LGE	Diffuse myocardium involvement	Rare; often associated with cardiomyopathy or other heart disease; needs further evaluation

LGE: Late Gadolinium Enchancement, LV: left ventricle.

**Table 3:** Summary table of studies, fibrosis patterns and clinical implications.

Study (First Author, Year)	Population	Fibrosis Detection Method	Prevalence of Fibrosis (%)	Fibrosis Pattern(s) Observed	Clinical Outcomes/Notes
Zorzi et al., 2016 [1]	Competitive endurance athletes (n = 54)	Cardiac MRI (LGE)	34%	Patchy (subepicardial/midmyocardial LV)	22% with patchy pattern had malignant arrhythmias; risk marker
Schnell et al., 2016 [4]	Asymptomatic endurance athletes (n = 36)	Cardiac MRI (LGE)	14%	Subepicardial delayed enhancement	Mostly benign, no arrhythmias reported
Kaddoura et al., 2025 [18]	Systematic review of athletes	Various (LGE-CMR predominant)	Variable	Patchy, diffuse patterns	Suggested subclinical fibrosis risk; need for longitudinal data
La Gerche et al., 2012 [8]	Endurance athletes (n = 40)	Echocardiography, biomarkers	N/A	Cardiac remodeling, fibrosis inferred	RV dysfunction, potential arrhythmogenic remodeling
Schmermund, 2018 [9]	Masters athletes (male) (n = 100+)	Coronary Computed tomography, LGE	CAC and fibrosis noted	Calcific and mixed plaques	Masters athletes may develop coronary atherosclerosis, requiring nuanced, evidence-based cardiovascular risk management.

CAC: Coronary Artery Calcium, CMR: Cardiac Magnetic Resonance, LGE: Late Gadolinium Enchancement, LV: left ventricle, MRI: magnetic resonance imaging, n: population, N/A: non applicable, RV: right ventricle.

**Table 4:** Management strategies for athletes with myocardial fibrosis.

Management Strategies	
**Exercise Restriction:**	In some cases, particularly those with high-risk features, exercise restriction may be recommended to reduce the risk of triggering arrhythmias or other adverse events. The degree of exercise restriction should be individualized based on the athlete’s risk profile [25].
**Medications:**	There is no solid evidence or guidelines [2,5,23].
**Implantable Cardioverter-Defibrillator (ICD):**	In athletes at high risk of life-threatening arrhythmias, an ICD should be considered on top of exercise restriction or reduction, to provide protection against sudden cardiac death.
**Regular Follow-up:**	Athletes with myocardial fibrosis should undergo regular follow-up with a cardiologist experienced in the management of athletes with cardiac conditions. Follow-up may include periodic Stress test, CMR, ECG, and echocardiography to monitor for any changes in their condition

CMR: Cardiac Magnetic Resonance, ECG: Electrocardiograph, ICD: Implantable Cardioverter Defibrillator.

## Data Availability

No new data were created or analyzed in this study.

## Abbreviations

The following abbreviations are used in this manuscript:

AF	Atrial Fibrillation
BNP	B-Type Natriuretic Peptide
CAC	Coronary Artery Calcium
CAD	Coronary Artery Disease
CMR	Cardiac Magnetic Resonance
ECG	Electrocardiograph
ECM	Extracellular Matrix
EF	Ejection Fraction
ICD	Implantable Cardioverter Defibrillator
IEE	Intense Endurance Exercise
LGE	Late Gadolinium Enhancement
LV	Left Ventricle
MACE	Major Adverse Cardiac Event
MI	Myocardial Infarction
MMPs	Matrix Metalloproteinases
MRI	Magnetic Resonance Imaging
NSVT	Non-Sustained Ventricular Tachycardia
RBBB	Right Bundle Branch Block
RV	Right Ventricle
TGF-β	Transforming Growth Factor-Beta
TIMPs	Tissue Inhibitors of Metalloproteinases
